# Supplementary material for: Characterization of the Putative Acylated Cellulose Synthase Operon in Komagataeibacter xylinus E25
Source: Int J Mol Sci. 2022 Jul 16;23(14):7851. doi: 10.3390/ijms23147851 (PMC9318390; doi:10.3390/ijms23147851)
Supplement: Supplementary file 1 [file ijms-23-07851-s001.zip › ijms-1819875-supplementary.pdf]

Characterization of the putative acylated cellulose synthase operon in *Komagataeibacter xylinus* E25

Izabela Szymczak[1+], Agnieszka J. Pietrzyk-Brzezińska[1+], Kajetan Duszyński[1], Małgorzata Ryngajłło[1*]

Institute of Molecular and Industrial Biotechnology, Lodz University of Technology, B. Stefanowskiego 2/22, 90-537 Lodz, Poland; izabela.redzynia@p.lodz.pl (ORCID: 0000-0002-0944-8237), agnieszka.pietrzyk-brzezinska@p.lodz.pl (ORCID: 0000-0003-1565-7307), kajetan.duszynski@dokt.p.lodz.pl, malgorzata.ryngajllo@p.lodz.pl (ORCID: 0000-0002-0430-0102)

*Correspondence: malgorzata.ryngajllo@p.lodz.pl; +48 0426313354
[+]These two authors contributed equally to the study.

SUPPLEMENTARY INFORMATION

Supplementary Tables

Table S1. Amino acid sequence identity (in %) between the protein products of *bcsII* operon of *K. xylinus* E25 and respective proteins of other *Komagataeibacter* type strains as well as homologs from bacteria that do not belong to *Acetobacteraceae* family (species representative sequences).

Strain name	BcsX	BcsY	BcsZ
K. swingsii LMG 22125[T]	100	98	97
K. intermedius TF2[T]	96	89	-
K. xylinus LMG 1515[T]	95	82	60
K. diospyri MSKU9[T]	94	88	-
K. sucrofermentans LMG 18788[T]	95	82	61
K. nataicola LMG 1536[T]	95	81	62
K. europaeus LMG 18890 [T]	90	95	91
K. pomaceti T5K1[T]	89	74	61
K. cocois WE7 [T]	89	74	61
K. maltaceti LMG 1592 [T]	87	74	62
K. xylilnus CGCMCC 2955	88	79	-
K. rhaeticus LMG 22126 [T]	87	79	-
K. saccharivorans LMG 1582 [T]	84	67	57
K. hansenii ATCC 53582	77	67	-
Ga. entanii LTH 4560 [T]	75	68	-
Polynucleobacter aenigmaticus hydrolase	46	-	-
Castellaniella defragrans hydrolase	42	-	-
Pseudomonas alcaligenes hydrolase	43	-	-
Enterobacter lignolyticus acyltransferase	-	52	-
Rhizobium sullae acyltransferase	-	36	-
Pseudomonas fluorescens acyltransferase	-	36	-
Caenorhabditis elegans acyltransferase	-	30	-
Bermanella marisrubri arylesterase	-	-	26
Desulfobacter hydrogenophilus arylesterase	-	-	30
Escherichia coli thioesterase	-	-	25

[T] Type strain

Table S2. Primer sequences used for PCR. Upper case letters represent the part of the sequence complementary to a gene, italic font was used to highlight restriction sites.

**Primer**	**Sequence**
bcsX_fw	5’-atat*ccatgg*CGATGAATGCCCTTCTTGCAGG-3’
bcsX_rv	5’-atat*ctcgag*ACCACGTCAGGATAGGCTTATTGC-3’
bcsY_fw	5’-atatat*caatgg*CGATGCTGCAACTTACCCCCTTC-3’
bcsY_rv	5’-atat*ctcgag*GGtTTAATGGACACGAGACACATGGG-3’
bcsZ_fw	5’-atatat*caatgg*CGATGCCGGGACGCCTG-3’
bcsZ_rv	5’-atat*ctcgag*TTATTTACTGAACAGGGTCCCGATAC-3’

Supplementary Figures

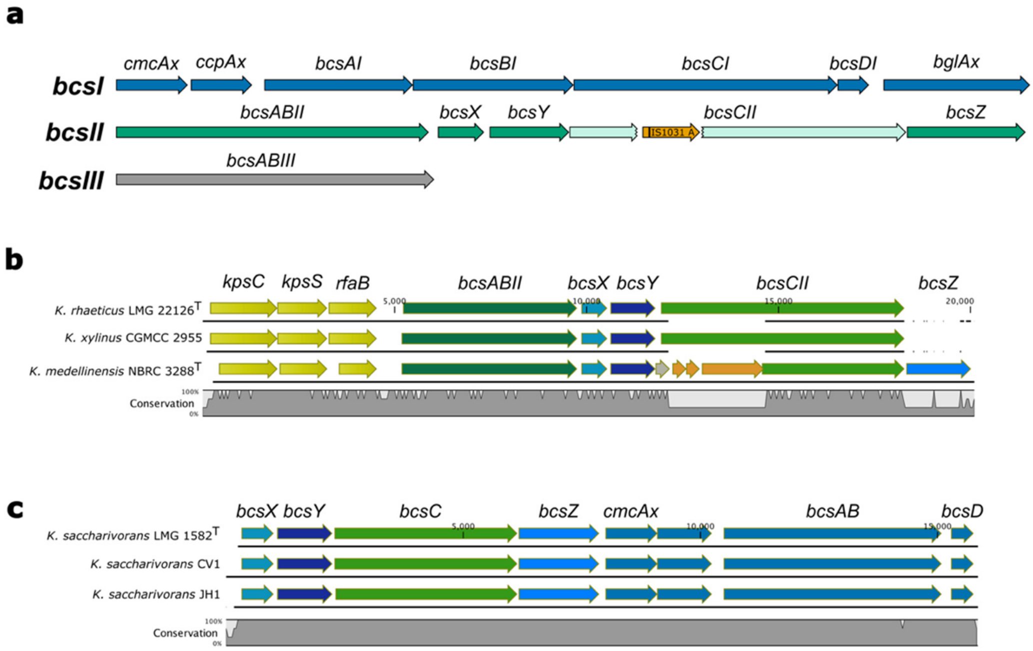

Figure S1. Cellulose synthase clusters in *K. xylinus* E25 and other *Komagataeibacter* species. a). operons *bcsI*, *bcsII* and *bcsIII* of *K. xylinus* E25; b). Alignment of operon *bcsII* in *K. rhaeticus* LMG 22126, *K. xylinus* CGMCC 2955, *K. medellinensis* NBRC 3288 with upstream cluster of genes associated with extracellular matrix formation (*kpsC, kpsS, rfaB*); c). Alignment of *bcsII* operon fused with *bcsI* in the strains of *K. saccharivorans*.

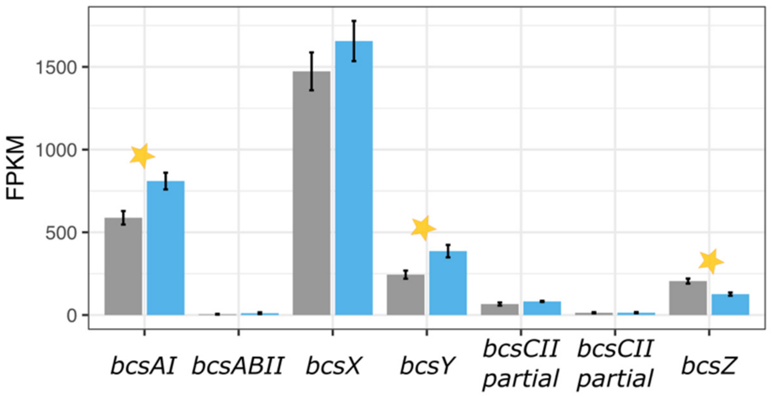

Figure S2. Changes in expression of the *bcs*AI gene and the *bcsII* operon based on published transcriptomic data for *K. xylinus* E25 (Ryngajłło et al., 2019). Genes are coloured according to $\log_2$ fold change in expression between SH and SH+EtOH cultures. Stars denote statistically significant changes (called by DESeq2; adjusted *p-value* ≤ 0.05). Thin black bars denote standard error.

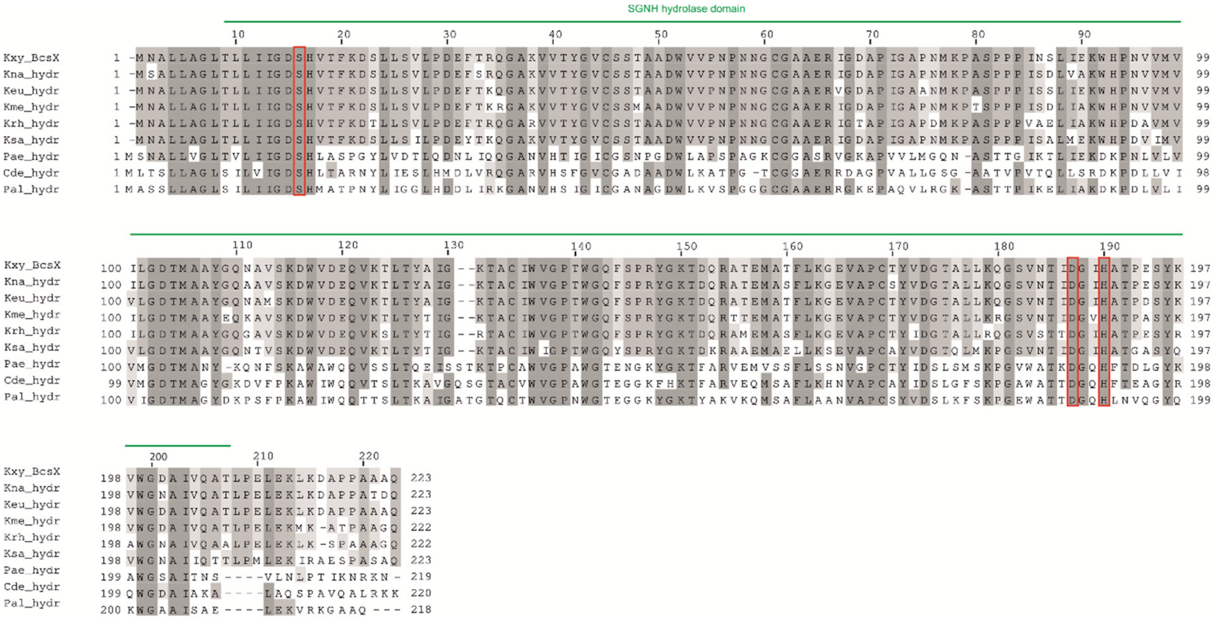

Figure S3. Sequence alignment of BcsX from *K. xylinus* E25 (Kxy) and SGNH hydrolases (hydr) from *K. nataicola* (Kna), *K. europaeus* (Keu), *K. medellinensis* (Kme), *K. rhaeticus* (Krh), *K. saccharivorans* (Ksa), *Polynucleobacter aenigmaticus* (Pae), *Castellaniella defragrans* (Cde), and *Pseudomonas alcaligenes* (Pal). Sequences were aligned using ClustalΩ (https://www.ebi.ac.uk/Tools/msa/clustalo) and presented using Jalview (http://www.jalview.org). Intensity of the gray color gradient reflects sequence identities and similarities. Numbering above the alignment is according to *K. xylinus* BcsX. The SGNH hydrolase domain is marked with a green line. The residues of the catalytic triad are presented in red boxes.

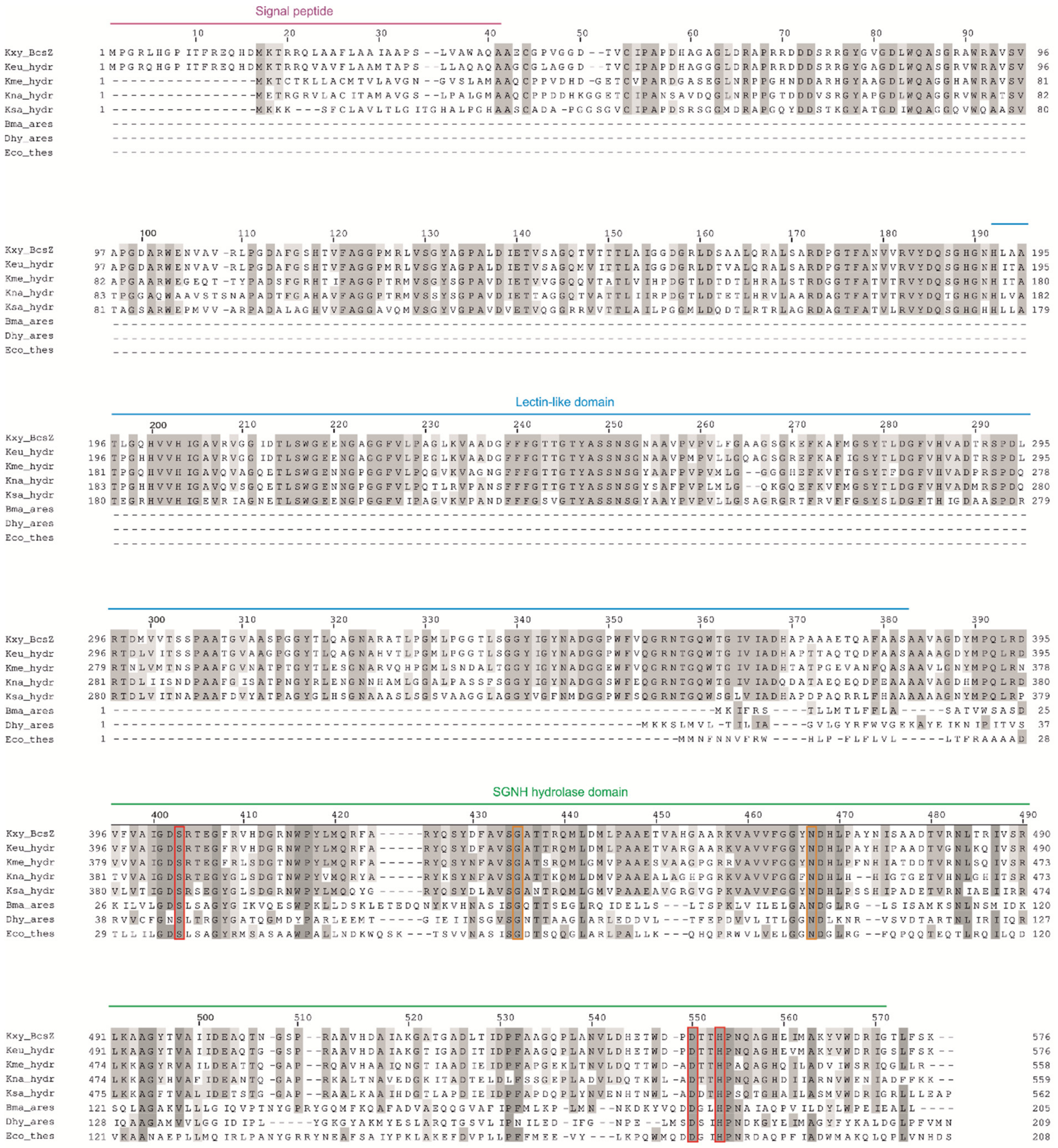

Figure S4. Sequence alignment of BcsZ from *K. xylinus* E25 (Kxy) and SGNH hydrolases (hydr) from *K. europaeus* (Keu), *K. medellinensis* (Kme), *K. nataicola* (Kna), *K. saccharivorans* (Ksa), arylesterases (ares) from *Bermanella marisrubri* (Bma), *Desulfobacter hydrogenophilus* (Dhy), and thioesterase (thes) from *Escherichia coli* (Eco). Sequences were aligned using ClustalΩ (https://www.ebi.ac.uk/Tools/msa/clustalo) and presented using Jalview (http://www.jalview.org). Intensity of the gray color gradient reflects sequence identities and similarities. Numbering above the alignment is according to *K. xylinus* BcsZ. Signal peptide, lectin-like domain and SGNH hydrolase domain are marked with a purple, blue and green line, respectively. The residues of the catalytic triad are presented in red boxes, whereas the residues of the oxyanion hole are in orange boxes.

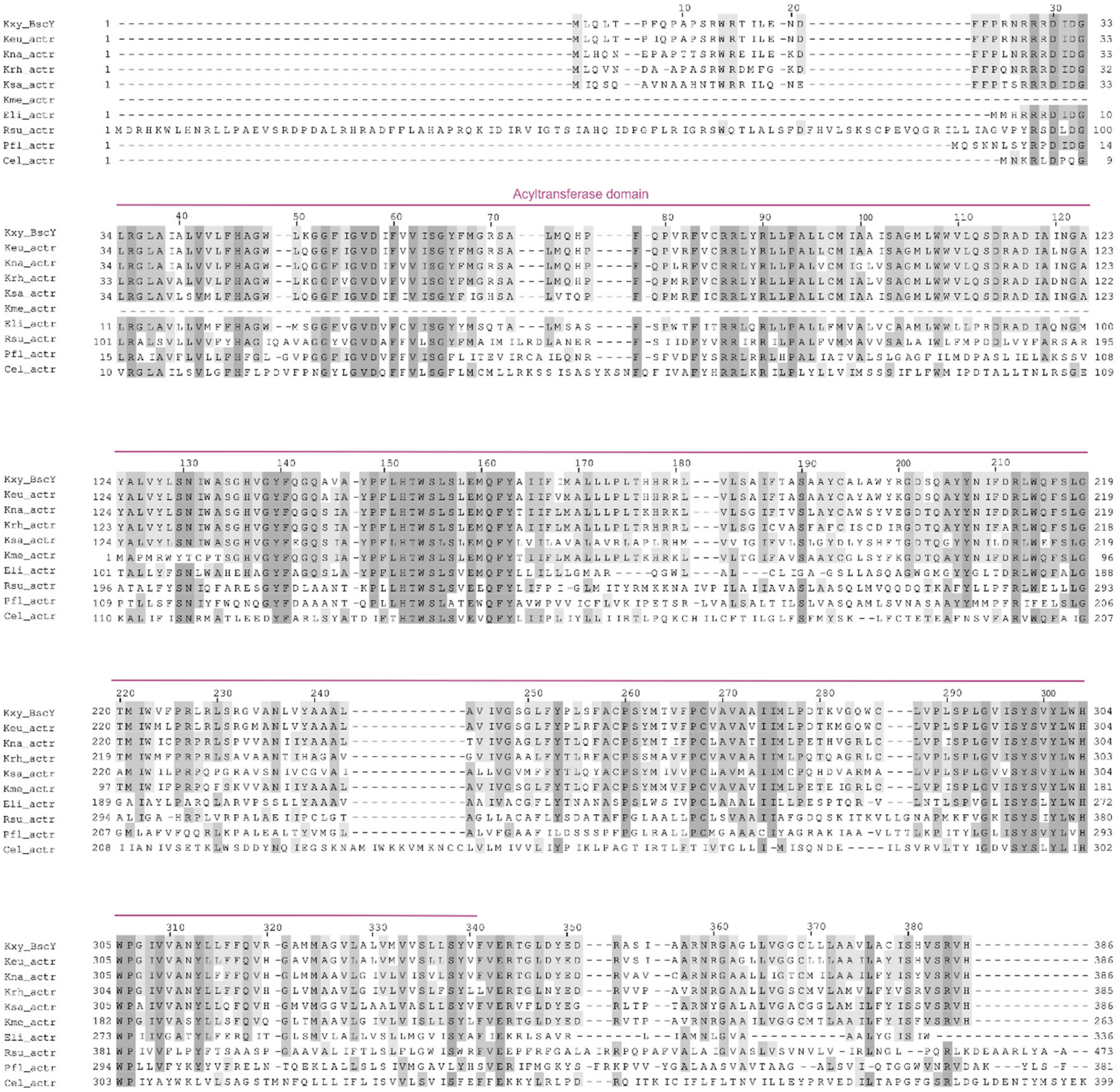

Figure S5. Sequence alignment of BcsY from *K. xylinus* E25 (Kxy) and acyltransferases (actr) from *K. europaeus* (Keu), *K. nataicola* (Kna), *K. rhaeticus* (Krh), *K. saccharivorans* (Ksa), *K. medellinensis* (Kme), *Enterobacter lignolyticus* (Eli), *Rhizobium sullae* (Rsu), *Pseudomonas fluorescens* (Pfl), and *Caenorhabditis elegans* (Cel). Sequences were aligned using ClustalΩ (https://www.ebi.ac.uk/Tools/msa/clustalo) and presented using Jalview (http://www.jalview.org). Intensity of the gray color gradient reflects sequence identities and similarities. Numbering above the alignment is according to *K. xylinus* BcsY. Acyltransferase domain is marked with a purple line.

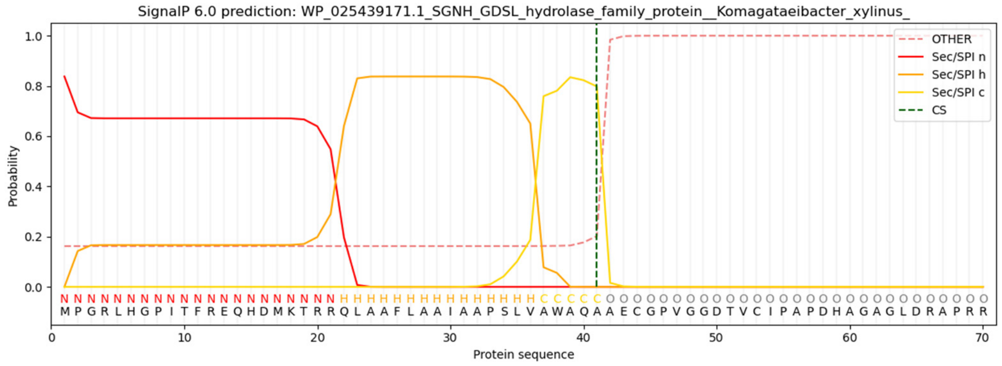

Figure S6. Prediction of signal peptide in the sequence of BcsZ from *K. xylinus* E25 by SignalP 6.0.

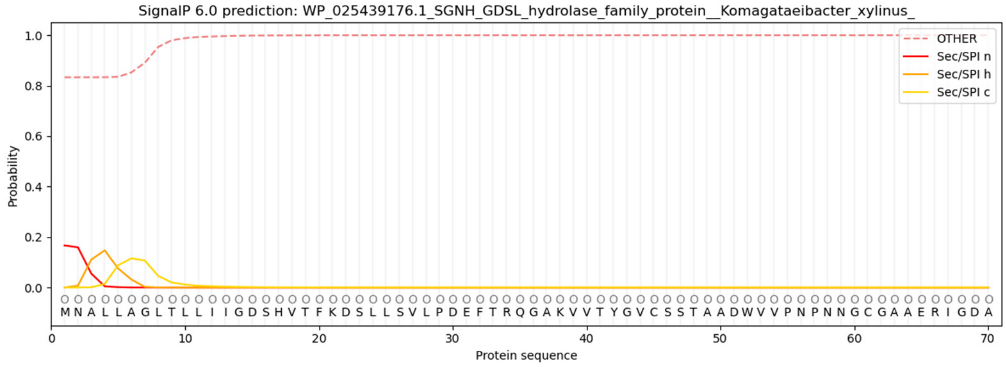

Figure S7. Prediction of signal peptide in the sequence of BcsX from *K. xylinus* E25 by SignalP 6.0.

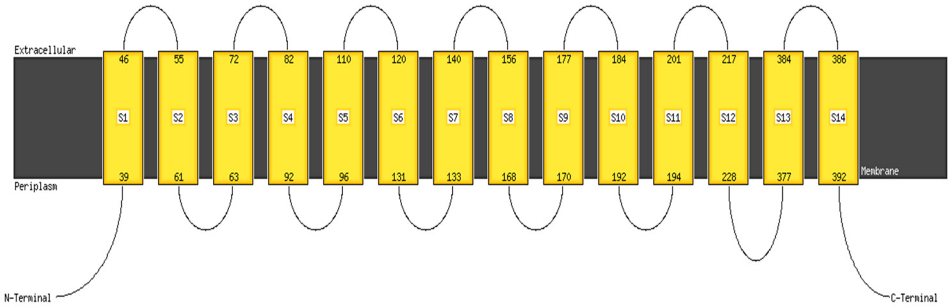

Figure S8. The predicted secondary structure elements of BcsY, the analysis was performed using PRED-TMBB2.
